# Autoantibodies against M_5_-muscarinic and beta_1_-adrenergic receptors in periodontitis patients

**DOI:** 10.18632/aging.103864

**Published:** 2020-08-28

**Authors:** Isabel Scherbaum, Harald Heidecke, Kübra Bunte, Ulrike Peters, Thomas Beikler, Fritz Boege

**Affiliations:** 1 Central Institute for Clinical Chemistry and Laboratory Diagnostics, Heinrich Heine University Düsseldorf, Medical Faculty, Düsseldorf, Germany; 2 Cell Trend GmbH, Luckenwalde, Germany; 3 Department of Periodontics, Preventive and Restorative Dentistry, University Medical Center Hamburg-Eppendorf, Hamburg, Germany

**Keywords:** parodontitis, autoantibodies, chronic heart failure, beta1-adrenergic receptor, M5-muscarinic receptor

## Abstract

Autoantibodies against muscarinic and beta_1_-adrenergic receptors are considered a potential cause and/or risk factor for chronic heart failure. Association of periodontitis with such autoantibodies and with impaired heart function has been observed in patients exposed to endemic Chagas' disease, which triggers by itself cardiomyopathy and receptor immunization.

Here we studied the association between periodontitis, markers of cardiac injury and receptor autoimmunization in periodontitis patients (n = 147) not exposed to Chagas' disease. The autoantibodies were determined by IgG binding to native intact muscarinic and beta_1_-adrenergic receptors or to a cyclic peptide mimicking the disease-relevant conformational autoepitope presented by the active beta_1_-adrenergic receptor. Possible cardiac injury and inflammatory status were judged by serum levels of proBNP/Troponin I and CRP/IL-6, respectively. These parameters were analysed in healthy and periodontally diseased individuals as well as before and after periodontal therapy.

Patients with periodontitis had significantly (p < 0.001) higher levels of autoantibodies against M_5_-muscarinic and beta_1_-adrenergic receptors, which further increased following periodontal therapy. Receptor autoantibodies were associated with increased inflammatory status but not with increased markers of cardiac injury. Thus, our data indicate that periodontitis triggers systemic inflammation, which is associated with receptor autoimmunization, and, independently thereof, with cardiac injury.

## INTRODUCTION

Periodontitis is a chronic inflammatory disease of the tooth-supporting tissues that leads to tooth loss if left untreated [1]. It is the sixth most common chronic disease, affecting up to 50% of the global population [2]. Oral dysbiosis, characterized by a microbial shift in favour of bacterial pathogens, initiates and perpetuates inflammatory and immunological dysregulations that cause the breakdown of periodontal tissues. However, these detrimental host responses are not only confined to the oral cavity but contribute to the pathogenesis of several other systemic diseases and conditions, e.g. diabetes, chronic kidney disease, obesity and cardiovascular diseases [3, 4].

Cardiovascular diseases (CVD) are the most common non-communicable diseases worldwide [5]. Periodontitis and cardiovascular diseases share common risk factors such as smoking and diabetes [6]. Moreover, periodontitis is suggested as an independent risk factor for CVD. In this regard, either direct effects, i.e. the invasion of periodontal bacteria into oral and non-oral tissues (such as atheroma plaques), and indirect effects, i.e. the increased production of inflammatory mediators like interleukin-6 and C-reactive protein have been found to contribute to the increased CVD risk in periodontitis patients [6–11].

Chronic heart failure (CHF) affects over 26 million people globally with an increasing prevalence [12]. Stimulatory autoantibodies against the adrenergic beta1-receptor subtype (β_1_AR-Aabs) and autoantibodies against muscarinic receptors (MR-Aabs) are frequently found in patients with CHF [13]. Periodontitis patients were reported to frequently exhibit circulating autoantibodies against the adrenergic beta1-receptor subtype (β_1_AR) associated with poorer cardiac function [14–16]. The presence of these antibodies is generally considered a relevant risk factor for CHF [17]. It is associated with poorer heart function [18] and prognosis [19] in non-ischemic chronic heart failure. Immunization against β_1_AR has been demonstrated to cause CHF compatible with human syndromes of non-ischemic chronic heart failure [20]. The presence of these autoantibodies and their cross-reactions with viral or bacterial proteins are suspected causes of a potentially cardiopathogenic autoimmunization. Furthermore, genetic predisposition, the immune and hormonal status, and a variety of environmental factors are thought to contribute to that autoimmune response [21]. However, the trigger mechanism remains unclear [17].

Periodontal disease is a plausible candidate for triggering autoimmunization against β_1_AR and muscarinic receptors (MR). Along that road, periodontitis could compromise heart function and induce CHF [7–10]. However, data that correlate periodontitis with a potentially cardiopathogenic autoimmunization against β_1_AR and MR are ambiguous. Previous reports of an association of periodontitis with high levels of β_1_AR and MR have been conducted in the South of Argentina, where a high prevalence of such autoimmunization is mainly attributed to the very high prevalence of endemic Chagas' disease [22]. Therefore, it is necessary to investigate the possible link between periodontitis, cardiac injury and autoimmunization against β_1_AR and muscarinic receptors under conditions where the confounding impact of endemic Chagas' disease can be excluded, i.e. a population living in mid-Europe. In the present study we follow up on this notion, assessing markers of cardiac injury and markers of inflammation in periodontitis patients and healthy controls and determining the impact of periodontal therapy on β_1_AR-Aabs and M_5_R-Aabs levels in a European population.

## RESULTS

**Figure 1:**
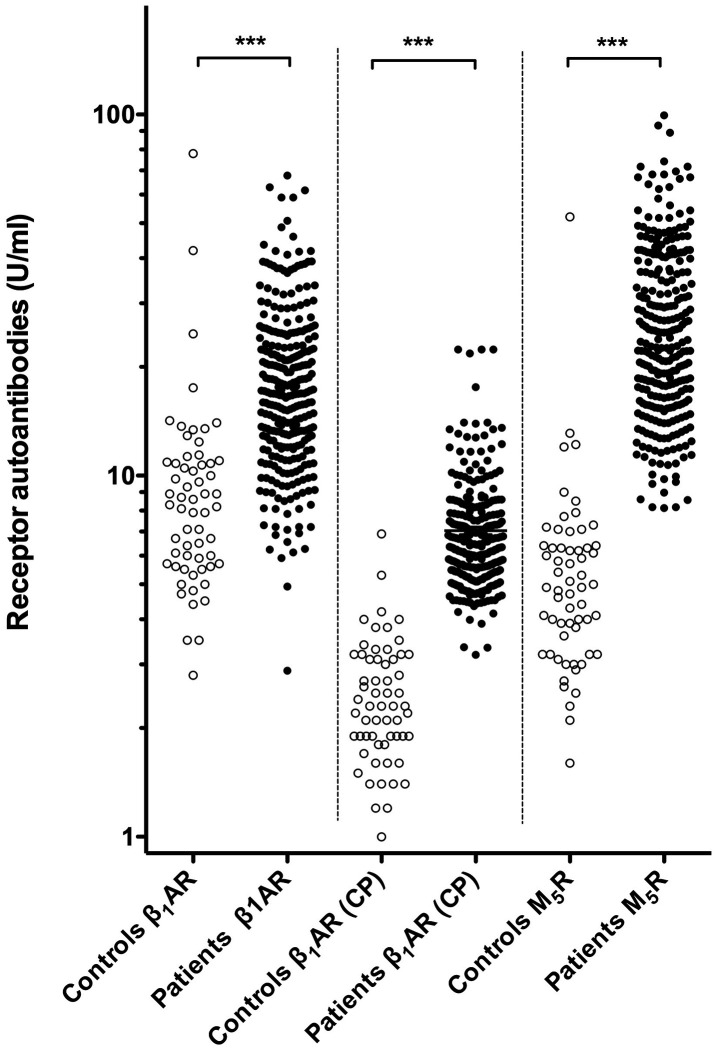
**Levels of circulating β_1_AR-Aabs and M_5_R-Aabs of patients (before therapy) and controls.** Left and Right: Serum levels of β_1_AR-Aabs and M_5_R-Aabs were measured by IgG-binding to the respective native receptors (CellTrend GmbH). Middle: β_1_AR-Aabs were determined by IgG-binding to a cyclic peptide providing a valid representation of the presumed pathogenic conformational auto-epitope within the second extracellular loop of the receptor associated with the active receptor conformation (indicated CP). ***: differences at p < 0.001 significance.

**Figure 2:**
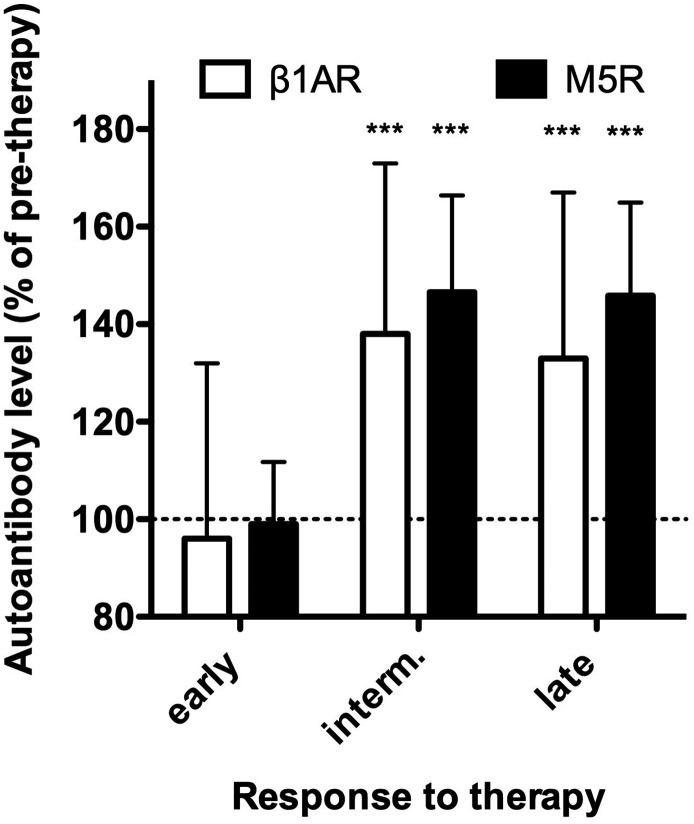
**Response of β_1_AR- and M_5_R-Aabs to therapy.** Serum levels of β_1_AR-Aabs (white) were measured by IgG-binding to a cyclic peptide representing the presumed pathogenic conformational auto-epitope within the second extracellular loop of the receptor. M_5_R-Aabs (black) were measured by IgG-binding to the native receptors (CellTrend GmbH). Values obtained at 5 and 17 weeks after therapy (early), 30 and 44 weeks after therapy (intermediate) and 58 to 112 weeks after therapy (late) are normalized to pre-therapeutic values (dotted line). Data of n=66 patients undergoing complete follow up are given as median ± interquartile range. *** indicate differences to pre-therapy values at p < 0.001 significance.

Before periodontal therapy, periodontitis patients exhibited statistically significant higher levels of β_1_AR-Aabs and M_5_R-Aabs than the healthy controls. Median β_1_AR-Aab levels as determined by IgG-binding to intact native receptor were about twice as high in patients not yet having undergone treatment (17.12 ± 10.02 Units/mL) as compared to healthy controls (8.22 ± 5.13 Units/mL). This difference was highly significant (p < 0.001), and could be confirmed by IgG-binding to a cyclic peptide representing the conformational auto-epitope within the second extracellular loop of the receptor associated with the active receptor conformation (Table 1). Recent evidence indicates that autoimmunity to this epitope is causally involved in the pathogenesis of dilated cardiomyopathy [23]. The latter, more specific assay revealed three-fold higher β_1_AR-Aab levels in patients not yet having undergone treatment (6.34 ± 2.55 ng/mL) as compared to healthy controls (2.31 ± 1.28 ng/mL) (p < 0.001). Moreover, serum levels of β_1_AR-Aabs (as determined by IgG-binding to the cyclopeptide) exhibited only a marginal overlap between controls and patients (Figure 1, middle). Given the apparent superior discriminative power of the cyclopeptide-assay, β_1_AR-Aab levels derived from this assay were selected for subsequent analyses (Table 2 and Figure 2). An even more pronounced difference between healthy controls and periodontitis patients was observed for the levels of circulating M_5_R-Aab. Periodontitis patients presented five-fold higher circulating M_5_R-Aab levels (24.14 ± 17.10 Units/mL) before periodontal therapy, compared to corresponding levels in healthy controls (4.90 ± 3.04 Units/mL) (p < 0.001). The values of circulating M_5_R-Aabs exhibited only a marginal overlap between controls and patients (Figure 1, d left). It should also be noted that in the patients, serum levels of M_5_R-Aabs and β_1_AR-Aabs were significantly correlated with each other (Supplementary Figure 2).

**Table 1:** Baseline characteristics of periodontitis patients and healthy individuals.

**Group Variables**	**Periodontitis (n=146)**	**Healthy (n=60)**	**P value^1^**
proBNP (ng/l)	49.46 (± 63.07)	35.33 (± 46.56)	= 0.002
TpI (ng/l)	3.30 (± 2.16)	3.00 (± 0.00)	< 0.001
CRP (mg/dl)	0.11 (± 0.22)	0.07(± 0.13)	= 0.160
IL-6 (ng/l)	1.60 (± 1.11)	1.50 (± 0.00)	< 0.001
β1AR-Aab^2^ (U/ml)	17.12 (± 10.02)	8.22 (± 5.13)	< 0.001
β1AR-Aab^3^ (ng/ml)	6.34 (± 2.55)	2.31 (± 1.28)	< 0.001
M_5_R-Aab^2^ (U/ml)	24.14 (± 17.10)	4.90 (± 3.04)	< 0.001
Gender (% female)	67.81	66.7	> 0.99
Age (years)	47 (± 16.11)	28 (± 11.66)	<0.001^4^

Median values of non-normally distributed data (± interquartile range)

^1^Significance of difference between groups

^2^IgG-binding to intact receptor

^3^IgG-binding to cyclopeptide representing the conformational auto-epitope within the second extracellular loop of the receptor

^4^Poor age-match of control group inevitable due to age-associated prevalence of periodontitis

**Table 2:** Correlation between receptor autoantibodies, cardiac markers and inflammation markers in periodontitis patients before and after therapy.

	**β_1_AR-Aab^1^**		**M_5_R-Aab^2^**	
	**Pre-therapy**	**Post-therapy^3^**	**Pre-therapy**	**Post-therapy^3^**
proBNP	-.042	.041	-.027	.044
TpI	-.024	.026	-,036	.035
CRP	.192*	.223*	.159	.195*
IL-6	.140	.280*	.074	.214*

Values given as correlation coefficients

*significance of correlation (p < 0.05)

^1^IgG-binding to cyclopeptide representing the conformational auto-epitope within the second extracellular loop of the receptor

^2^IgG-binding to intact receptor

^3^Five weeks or more, all follow ups summarized

In the next step, we examined the impact of periodontal therapy on β_1_AR and M_5_R autoimmunization. For this purpose, we compared within the periodontitis group baseline levels of β_1_AR- and M_5_R-Aabs (pre-therapy and on the day of therapy) with corresponding values measured at each of the post-therapy follow-up visits. Autoantibody levels did not significantly change from pre-therapy to follow-up determinations at 8 and 17 weeks after therapy. However, at 30 weeks and more after therapy, β_1_AR- and M_5_R-Aabs were significantly (p < 0.001) increased by about 40% above pre-therapy levels, and these increases remained stable until 112 weeks after therapy (Figure 2). It should be noted that data from all patients, who left the study at any time during post-therapy follow-up for undisclosed reasons (N = 80) were excluded from the above longitudinal analyses.

In the next step, we addressed the link between periodontitis, cardiac injury and generalized inflammation suggested by various studies, e.g. [24]. For that purpose, we measured the serum-levels of cardiac (proBNP, TpI) and inflammatory markers (CRP, IL-6) and compared these values between healthy individuals and periodontitis patients in pre-therapy samples. Median values of cardiac markers were significantly higher in the periodontitis group compared to the control (see Table 1). Likewise, the periodontitis group showed significantly higher serum levels of IL-6, while the CRP levels did not differ significantly between the groups (Table 1). In summary, these results confirm previous studies [6–11, 25, 26]. In addition, we noticed that in post-therapy follow up of periodontitis patients (5 weeks and more) TpI increased (by 8.5%, p < 0.001). proBNP exhibited a similar, albeit insignificant, trend to increase during post-therapy follow. In contrast, the inflammation markers exhibited a weak and insignificant trend to decrease upon therapy (not shown).

Finally, we addressed the question whether cardiac injury, generalized inflammation and autoimmunization against β_1_AR and/or M_5_R were interrelated or at least co-incident in the patients. To test this hypothesis, we analyzed correlations between the levels of the cardiac and inflammatory markers and the serum levels of circulating β_1_AR-Aabs or M_5_R-Aabs. These data are summarised in Table 2. β_1_AR-Aabs and M_5_R-Aabs were significantly correlated with the level of inflammation markers. Interestingly, this correlation was much more pronounced during therapy follow-up. In contrast, β_1_AR- and M_5_R-Aabs were not correlated with the cardiac markers (neither before nor after therapy). In summary these findings suggest that receptor-autoimmunization is a by-product of the inflammatory response to periodontitis (and the therapy thereof), but not directly associated with the extent of cardiac injury in these patients.

## DISCUSSION

This study was carried out, in order to investigate the impact of periodontitis and its treatment on the prevalence of circulating of β_1_AR-Aab and to address the possible involvement of these autoantibodies in coincident cardiac injury. Autoantibodies against MR were included in the study, given the involvement of such autoantibodies in many autoimmune-diseases including CHF (see below). We focused here on autoantibodies against the M_5_-subtype, because among all muscarinic receptor subtypes tested, M_5_R-autoantibodies exhibited the strongest association with the decline of cardiac function in a cohort of post-myocarditis patients (unpublished results from a prospective cohort study on etiology and titer-course of cardiac autoantibodies and their effect *on* survival (ETiCS-study) [27], mentioned with kind permission of the study coordinator). Moreover, we observed in pre-experiments that M_5_R-Aabs levels exhibited a clear difference between periodontitis patients and healthy controls.

### Salient findings

We confirm previous reports [14–16] of an association between periodontitis and circulating β_1_AR-Aabs. We could exclude the suspected confounding impact of endemic Chagas' disease on the previously mentioned study. Beyond that, we observed that periodontitis is also, and even more clearly, associated with increased levels of M_5_R-Aabs. When drawing that conclusion, it must, however, be taken into account that the control group was much younger, which is inevitable due to the high prevalence of periodontitis in the elderly. Since autoimmune status changes with age, it is difficult to conclude that the higher levels of autoantibodies in the patients are solely due the disease. Moreover, β_1_AR-Aabs and M_5_R-Aabs were highly coincident (i.e. were increased in the same patients, see Supplementary Figure 2) in the periodontitis patients of our cohort and serum levels of both autoantibody species further increased upon periodontal therapy. Our data support previous reports of an association of periodontitis with systemic inflammation and impaired cardiac function. However, there is no indication that an enhanced receptor-autoimmunity is linked to increases in serum markers of cardiac injury, which is the case in other etiologies of CVD. Thus, increased prevalence of β_1_AR-Aabs and in particular of M_5_R-Aabs could be a distinct feature of periodontitis and periodontitis-associated autoimmunization against these two receptors, which is possibly boosted upon periodontal therapy. However, these phenomena may not directly be associated with increases in serum markers of cardiac injury inferring a direct link to impaired cardiac function.

### Possible links between periodontitis and receptor autoimmunization

Chagas' disease is the model for cardio-pathogenic receptor autoimmunization. In this disease immunization against the ribosomal P2beta protein of *T. cruzi* induces humoral autoimmunity against the β_1_AR, and the β_1_AR-Aabs thus induced are the cause of CVD occurring 10-20 after infection with *T cruzi* [28]. Interestingly, Chagas’ disease is only seen in the western hemisphere. It still remains unclear what triggers receptor-directed humoral autoimmune responses in CVD unrelated to Chagas' disease [17]. It has been suspected for a very long time that the trigger could be bacterial or viral antigens [29]. On the other hand, it is known that the invasive interventions in periodontal therapy entail release of bacterial antigens into the blood stream on a large scale [6]. Consequently, it is tempting to speculate that such exposure of the immune system to periodontal bacteria and increased circulating proinflammatory markers could be the cause of the high prevalence of autoimmunization against β_1_AR and M_5_R observed in periodontitis. The changes in circulating levels of β_1_AR- and M_5_R-Aabs after therapy seen here are in line with this notion, as serum levels of receptor autoantibodies remained stable immediately after therapy, but increased within several weeks post-therapy, which is consistent with the typical time window of immuniszation e.g. vaccination. Serum levels of receptor autoantibodies were highest in the intermediate observation window, in which follow-up frequency (and associated re-exposure of the immune system to periodontal bacteria) was the highest (every three months). The levels decreased again in the late observation window when the follow-up frequency was reduced to twice a year. In summary, these observations indicate that repeated release of bacterial antigens and the periodontitis-associated release of inflammatory markers into the blood stream possibly induce and maintain autoimmunization against β_1_AR and M_5_R in periodontitis patients. This notion is also supported by the apparent correlation between post-therapy levels of receptor autoantibodies and inflammation markers.

### Etiological implications of M_5_R-Aabs

Autoantibodies against muscarinic acetylcholine receptors have been implied in many diseases. Autoantibodies against the M_2_ subtype play a role in a variety of cardiovascular diseases [30–39]. Autoantibodies against the M_3_-subtype are involved in Sjögren's syndrome [40]. Autoantibodies against the M_3_- and M_4_-subtypes cause the chronic fatigue syndrome [41, 42]. Autoantibodies against the M_5_-subtype have rarely been observed in the context of diseases. The only published disease-associated incidence of M_5_R-Aabs has been reported from a small subgroup of female patients suffering from postural orthostatic tachycardia syndrome. These patients also exhibited increased levels of autoantibodies against all other MR-subtypes and various adrenergic receptors [43]. Interestingly, in an ongoing prospective cohort study on titer-course *of cardiac autoantibodies and their effect on* survival (ETiCS-study) [27], which included analysis of autoantibodies against all MR subtypes, the M_5_R-autoantibodies exhibited the strongest association with decline of cardiac function (unpublished results mentioned with kind permission of the study coordinator). The M_5_R plays a crucial role in cholinergic dilation of the microcirculation most notably of the cerebrum [44, 45]. In mice, loss of this receptor leads to cognitive deficits [46]. If periodontitis-associated autoantibodies had the potency to inhibit or stimulate the M_5_R (yet to be confirmed), one of their most probable biological effect would be an interference with the regulation of cerebral microcirculation. Interestingly, autoantibodies against various other regulatory receptors of cerebral microcirculation have been implicated in the pathogenesis of Alzheimer's disease and vascular dementia in humans [47]. Moreover, large population-based association studies suggest a link between periodontitis and dementia [48]. Thus, M_5_R-Aabs could provide a mechanistic link between periodontitis and the associated risk of vascular dementia, therefore it may be of high value to follow up on this marker in future population-based studies.

### Etiological implications of β_1_AR-Aabs

The potential of β_1_AR-Aabs to cause or promote cardiovascular pathogenesis is firmly established by the Chagas' disease paradigm, as well as animal models of passive and active immunization and clinical therapy trials of removal and/or neutralization of these autoantibodies [17]. A variety of mechanisms have been demonstrated by which β_1_AR-Aabs can possibly harm the cardiovascular system [49]. Current belief holds that the common denominator of cardiotoxicity of β_1_AR-Aabs is the stabilization of an active conformation of the β_1_AR-molecule, entailing a chronic stimulation and/or hyper-sensitization of β_1_-adrenergic signal transduction in the heart [50] and related target tissues [49].

In the present study, we investigate another example of an epidemiological association between cardiac injury (evidenced by an increase in serum proBNP) and increased serum levels of β_1_AR-Aabs. The increased levels of β_1_AR-Aabs levels detected in case of periodontitis plausibly belong to the cardio-noxious variety, because β_1_AR-Aabs bind to a cyclic peptide, which was recently demonstrated to mimic the conformational epitope related to the active conformation of the receptor molecule, which constitutes the cardio-pathogenic autoepitope [23]. Nevertheless, our data do not support the conclusion that these autoantibodies are indeed the cause of the observed increases in cardiac markers (indicating cardiac injury and inferring impaired cardiac function in the periodontitis patients), because increased circulating levels of β_1_AR-Aabs (reacting with the presumed cardio-pathogenic autoepitope) in the periodontitis group were not correlated to increased levels of cardiac markers. Interestingly, this lack of correlation was also observed following therapy although median levels of both cardiac markers and β_1_AR-Aabs increased upon therapy. Thus, it seems plausible to assume that receptor-autoimmunization and cardiac injury are independent consequences of enhanced inflammation. One must also take into consideration that age distribution in the periodontitis group was much higher than in the control group. Therefore, the poorer cardiovascular function apparent in the patients may at least in part be due to age-related risk factors [8, 51].

### Practical conclusions

Currently, monitoring of periodontitis relies mostly on the mechanical probing of dental pockets, followed by mechanical debridement of the intraoral hard and soft tissues. The association between periodontitis and CVD, that is evidenced by multiple studies [6–11] and supported here by the observation of associated increases in serum markers of cardiac injury, is to our knowledge only sparsely monitored or even considered in routine dental healthcare. Conversely, monitoring of cardiac function in the elderly does not stringently include surveillance of the periodontal status. Several studies suggest that screening and therapy of periodontitis and cardiac function should be coordinated in the elderly [6–11, 25, 26]. The data presented here confirm this notion. We suggest that coordinated surveillance of CVD and periodontitis could *inter alia* be achieved by introducing determinations of serum proBNP into standard dental healthcare. Conversely, M_5_R-Aabs could provide a distinct serological marker of periodontitis, which in clinical settings not encompassing dental care possibly would allow for a preliminary stratification of periodontitis risk. Most notably, it should be considered to evaluate M_5_R-Aabs as a possible marker for the risk of vascular dementia in the context of periodontitis.

## MATERIALS AND METHODS

### Study participants

Study subjects were recruited from a prospective cohort study that included 147 initially untreated periodontitis patients at University of Münster, Germany. Periodontitis was diagnosed using case definitions in population-based studies [52] in accordance with the previously used 1999 Workshop classification [53]. 60 systemically and periodontally healthy individuals were recruited as controls at University of Düsseldorf. Absence of periodontitis was determined by probing pocket depths ≤ 2mm.

The study was performed in accordance with the Declaration of Helsinki and was approved by the Institutional Review Boards of University of Münster (IRB approval Nr. 1VBei) and University of Düsseldorf (IRB approval Nr. 3786). All participants have given their written consent to participate, were residents in Germany and none of them had a history of Chagas' disease and/or lived for the past 20 years in areas, where Chagas' disease is endemic. Exclusion criteria included the use of systemic antibiotics within six months prior to study enrolment, requirement of antibiotic prophylaxis, history of endocarditis, bleeding disorders, history of organ transplantation, dialysis, pregnancy and lactation.

### Study design and sampling

The healthy group was subjected to a one-time supragingival debridement and blood sample collection at the time of recruitment. Periodontitis group also received supragingival debridement at the time of recruitment- Supragingival debridement was followed with non-surgical periodontal therapy after 6-8 weeks. Non-surgical periodontal therapy consisted of supra- and subgingival debridement of oral hard and soft tissues, and adjunctive antimicrobial therapy with 0.2% chlorhexidin-containing mouthrinse used three times daily for 10 days. Surgical periodontal therapy and tooth extractions were performed 10-14 weeks after non-surgical periodontal therapy. The periodontitis group was followed up for a total period of two years after non-surgical therapy. The follow-up was done every 3 months during the first year and every 6 months during the second year. Follow-up examinations comprised of a comprehensive oral examination, supportive periodontal therapy (supra- und subgingival debridement) and blood sample collection. Baseline values of periodontitis group were derived from two samplings carried out at 2 weeks before non-surgical periodontal therapy (hereafter referred to as pre-therapy) and on the day of non-surgical periodontal therapy (referred to as therapy). Data of early therapy responses were collected from two samplings carried out 5 and 17 weeks after therapy. Intermediate responses were derived from two subsequent samplings 30 and 44 weeks after therapy. Late responses were summarised from subsequent samplings at 58 to 112 weeks after therapy. A total of 50 ml venous blood was collected from each participant by antecubital vein puncture at each visit.

### Laboratory tests

Pre-analytical handling of blood samples and determination of established parameters of generalized inflammation (CRP and IL-6), myocardial ischemia (TpI), and cardiac wall tension (proBNP) in sera followed routine diagnostic procedures accredited according to DIN EN ISO 15689. β_1_AR-Aabs, and autoantibodies against the muscarinic acetylcholine receptor M_5_ (M_5_R-Aabs), were measured with commercially available ELISAs (CellTrend GmbH, Luckenwalde, Germany) according to the instructions of the manufacturer. Both these assays provide native receptors presented in their physiological membrane environment as immunogenic targets for IgG binding. In addition, β_1_AR-Aabs were determined by IgG-binding to a cyclic peptide providing a valid representation of the conformational epitope within the second extracellular loop of the receptor associated with the active receptor conformation. It has been demonstrated that pre-absorption with this peptide neutralises the cardio-pathogenic potency of stimulatory receptor antibodies in mice [23]. The cyclic peptide was coated onto microtiter plates by established procedures and these plates were processed in a similar manner as the above commercial assays. The two assays for β_1_AR-Aabs exhibited a reasonable correlation (Suppl. Figure 1) with just a few extreme outliers, which, most probably, are due to the presence of β_1_AR-Aabs not directed against the second extracellular loop of the receptor.

### Statistical methods

All data analyses were performed using IBM SPSS Statistics 26.0 software (IBM Corp. Released 2019. IBM SPSS Statistics for Windows, Version 26.0. Armonk, NY: IBM Corp.). Normal distribution was tested by the method of Shapiro-Wilk. Median values and interquartile range (median ± IQR) are stated, when parameters exhibited non-normal distribution. Otherwise mean values and standard deviation (mean ± SD) are stated. The Mann-Whitney-U test was used to analyze differences between baseline values of periodontitis patients (before therapy) and controls. Friedman's test was used to detect an influence of periodontal therapy on β_1_AR and M_5_R autoimmunization and post-hoc tests with Bonferroni correction were carried out to determine which therapy time points differed significantly. Wilcoxon's signed rank-test was used for longitudinal analyses of the patient group before and after therapy. Spearman’s correlation was performed to compare methods and assess parameter correlations at pre- and post-therapy within the periodontitis group. Bivariate comparisons were performed to avoid increased type II error probability arising from multiple comparisons with adjusted p values. All tests were performed with the standard 0.05 level of statistical significance.

## Supplementary Material

Supplementary Figures

## References

[1] Pihlstrom BL, Michalowicz BS, Johnson NW. Periodontal diseases. Lancet. 2005; 366:1809–20. 10.1016/S0140-6736(05)67728-8 16298220

[2] Kassebaum NJ, Bernabé E, Dahiya M, Bhandari B, Murray CJ, Marcenes W. Global burden of severe periodontitis in 1990-2010: a systematic review and meta-regression. J Dent Res. 2014; 93:1045–53. 10.1177/0022034514552491 25261053 PMC4293771

[3] Chapple IL, Genco R, and Working group 2 of joint EFP/AAP workshop. Diabetes and periodontal diseases: consensus report of the joint EFP/AAP workshop on periodontitis and systemic diseases. J Clin Periodontol. 2013 (Suppl 14); 40:S106–12. 10.1111/jcpe.12077 23627322

[4] Linden GJ, Herzberg MC, and Working group 4 of joint EFP/AAP workshop. Periodontitis and systemic diseases: a record of discussions of working group 4 of the joint EFP/AAP workshop on periodontitis and systemic diseases. J Clin Periodontol. 2013 (Suppl 14); 40:S20–23. 10.1111/jcpe.12091 23627330

[5] WHO CVD Risk Chart Working Group. World health organization cardiovascular disease risk charts: revised models to estimate risk in 21 global regions. Lancet Glob Health. 2019; 7:e1332–45. 10.1016/S2214-109X(19)30318-3 31488387 PMC7025029

[6] Sanz M, Marco Del Castillo A, Jepsen S, Gonzalez-Juanatey JR, D’Aiuto F, Bouchard P, Chapple I, Dietrich T, Gotsman I, Graziani F, Herrera D, Loos B, Madianos P, et al. Periodontitis and cardiovascular diseases: consensus report. J Clin Periodontol. 2020; 47:268–88. 10.1111/jcpe.13189 32011025 PMC7027895

[7] Hansen GM, Egeberg A, Holmstrup P, Hansen PR. Relation of periodontitis to risk of cardiovascular and all-cause mortality (from a danish nationwide cohort study). Am J Cardiol. 2016; 118:489–93. 10.1016/j.amjcard.2016.05.036 27372888

[8] Dietrich T, Sharma P, Walter C, Weston P, Beck J. The epidemiological evidence behind the association between periodontitis and incident atherosclerotic cardiovascular disease. J Periodontol. 2013; 84:S70–84. 10.1902/jop.2013.134008 23631585

[9] Lockhart PB, Bolger AF, Papapanou PN, Osinbowale O, Trevisan M, Levison ME, Taubert KA, Newburger JW, Gornik HL, Gewitz MH, Wilson WR, Smith SC Jr, Baddour LM, and American Heart Association Rheumatic Fever, Endocarditis, and Kawasaki Disease Committee of the Council on Cardiovascular Disease in the Young, Council on Epidemiology and Prevention, Council on Peripheral Vascular Disease, and Council on Clinical Cardiology. Periodontal disease and atherosclerotic vascular disease: does the evidence support an independent association?: a scientific statement from the American Heart Association. Circulation. 2012; 125:2520–44. 10.1161/CIR.0b013e31825719f3 22514251

[10] Blaizot A, Vergnes JN, Nuwwareh S, Amar J, Sixou M. Periodontal diseases and cardiovascular events: meta-analysis of observational studies. Int Dent J. 2009; 59:197–209. 19774803

[11] Bahekar AA, Singh S, Saha S, Molnar J, Arora R. The prevalence and incidence of coronary heart disease is significantly increased in periodontitis: a meta-analysis. Am Heart J. 2007; 154:830–37. 10.1016/j.ahj.2007.06.037 17967586

[12] Bui AL, Horwich TB, Fonarow GC. Epidemiology and risk profile of heart failure. Nat Rev Cardiol. 2011; 8:30–41. 10.1038/nrcardio.2010.165 21060326 PMC3033496

[13] Bornholz B, Roggenbuck D, Jahns R, Boege F. Diagnostic and therapeutic aspects of β1-adrenergic receptor autoantibodies in human heart disease. Autoimmun Rev. 2014; 13:954–62. 10.1016/j.autrev.2014.08.021 25149394

[14] Segovia M, Ganzinelli S, Reina S, Borda E, Sterin-Borda L. Role of anti-β1 adrenergic antibodies from patients with periodontitis in cardiac dysfunction. J Oral Pathol Med. 2012; 41:242–48. 10.1111/j.1600-0714.2011.01090.x 21958237

[15] Segovia M, Reina S, Borda E, Sterin-Borda L. Autoantibodies to the β_1_-adrenoceptor from patients with periodontitis as a risk factor for cardiac dysfunction. ISRN Dent. 2011; 2011:791393. 10.5402/2011/791393 21991485 PMC3170702

[16] Reina S, Ganzinelli S, Sterin-Borda L, Borda E. Pro-apoptotic effect of anti-β_1_-adrenergic receptor antibodies in periodontitis patients. Int Immunopharmacol. 2012; 14:710–21. 10.1016/j.intimp.2012.10.011 23103827

[17] Boivin-Jahns V, Jahns R. GPCR-autoantibodies in chronic heart failure. Front Biosci (Landmark Ed). 2018; 23:2065–81. 29772546 10.2741/4690

[18] Jahns R, Boivin V, Siegmund C, Inselmann G, Lohse MJ, Boege F. Autoantibodies activating human β_1_-adrenergic receptors are associated with reduced cardiac function in chronic heart failure. Circulation. 1999; 99:649–54. 10.1161/01.cir.99.5.649 9950662

[19] Störk S, Boivin V, Horf R, Hein L, Lohse MJ, Angermann CE, Jahns R. Stimulating autoantibodies directed against the cardiac β_1_-adrenergic receptor predict increased mortality in idiopathic cardiomyopathy. Am Heart J. 2006; 152:697–704. 10.1016/j.ahj.2006.05.004 16996841

[20] Jahns R, Boivin V, Hein L, Triebel S, Angermann CE, Ertl G, Lohse MJ. Direct evidence for a β_1_-adrenergic receptor-directed autoimmune attack as a cause of idiopathic dilated cardiomyopathy. J Clin Invest. 2004; 113:1419–29. 10.1172/JCI20149 15146239 PMC406525

[21] Nussinovitch U, Shoenfeld Y. The clinical significance of anti-beta-1 adrenergic receptor autoantibodies in cardiac disease. Clin Rev Allergy Immunol. 2013; 44:75–83. 10.1007/s12016-010-8228-9 21188649

[22] Muñoz-Saravia SG, Haberland A, Wallukat G, Schimke I. Chronic chagas’ heart disease: a disease on its way to becoming a worldwide health problem: epidemiology, etiopathology, treatment, pathogenesis and laboratory medicine. Heart Fail Rev. 2012; 17:45–64. 10.1007/s10741-010-9211-5 21165698

[23] Wölfel A, Sättele M, Zechmeister C, Nikolaev VO, Lohse MJ, Boege F, Jahns R, Boivin-Jahns V. Unmasking features of the auto-epitope essential for β_1_-adrenoceptor activation by autoantibodies in chronic heart failure. ESC Heart Fail. 2020; 7:1830–41. 10.1002/ehf2.12747 32436653 PMC7373925

[24] Schulze-Späte U, Mizani I, Salaverry KR, Chang J, Wu C, Jones M, Kennel PJ, Brunjes DL, Choo TH, Kato TS, Mancini D, Grbic J, Schulze PC. Periodontitis and bone metabolism in patients with advanced heart failure and after heart transplantation. ESC Heart Fail. 2017; 4:169–77. 10.1002/ehf2.12126 28451454 PMC5396042

[25] Schenkein HA, Loos BG. Inflammatory mechanisms linking periodontal diseases to cardiovascular diseases. J Periodontol. 2013; 84:S51–69. 10.1902/jop.2013.134006 23631584

[26] Aarabi G, Heydecke G, Seedorf U. Roles of oral infections in the pathomechanism of atherosclerosis. Int J Mol Sci. 2018; 19:1978. 10.3390/ijms19071978 29986441 PMC6073301

[27] Deubner N, Berliner D, Schlipp A, Gelbrich G, Caforio AL, Felix SB, Fu M, Katus H, Angermann CE, Lohse MJ, Ertl G, Störk S, Jahns R, and Etiology, Titre-Course, and Survival-Study Group. Cardiac β_1_-adrenoceptor autoantibodies in human heart disease: rationale and design of the etiology, titre-course, and survival (ETiCS) study. Eur J Heart Fail. 2010; 12:753–62. 10.1093/eurjhf/hfq072 20494925

[28] Lopez Bergami P, Gómez KA, Levy GV, Grippo V, Baldi A, Levin MJ. The beta1 adrenergic effects of antibodies against the c-terminal end of the ribosomal P2beta protein of trypanosoma cruzi associate with a specific pattern of epitope recognition. Clin Exp Immunol. 2005; 142:140–47. 10.1111/j.1365-2249.2005.02885.x 16178868 PMC1809475

[29] Hoebeke J. Structural basis of autoimmunity against G protein coupled membrane receptors. Int J Cardiol. 1996; 54:103–11. 10.1016/0167-5273(96)02586-7 8803673

[30] Gurses KM, Yalcin MU, Kocyigit D, Kesikli SA, Canpolat U, Yorgun H, Sahiner ML, Kaya EB, Hazirolan T, Ozer N, Oto MA, Guc D, Aytemir K. M2-muscarinic acetylcholine receptor autoantibody levels predict left atrial fibrosis severity in paroxysmal lone atrial fibrillation patients undergoing cryoablation. Europace. 2015; 17:239–46. 10.1093/europace/euu228 25238749

[31] Yoshizawa A, Nagai S, Baba Y, Yamada T, Matsui M, Tanaka H, Miyoshi S, Amagai M, Yoshikawa T, Fukuda K, Ogawa S, Koyasu S. Autoimmunity against m₂muscarinic acetylcholine receptor induces myocarditis and leads to a dilated cardiomyopathy-like phenotype. Eur J Immunol. 2012; 42:1152–63. 10.1002/eji.201142104 22328321

[32] Pei J, Li N, Chen J, Li X, Zhang Y, Wang Z, Zhang P, Cao K, Pu J. The predictive values of beta1-adrenergic and M2 muscarinic receptor autoantibodies for sudden cardiac death in patients with chronic heart failure. Eur J Heart Fail. 2012; 14:887–94. 10.1093/eurjhf/hfs082 22713286

[33] Stavrakis S, Kem DC, Patterson E, Lozano P, Huang S, Szabo B, Cunningham MW, Lazzara R, Yu X. Opposing cardiac effects of autoantibody activation of β-adrenergic and M2 muscarinic receptors in cardiac-related diseases. Int J Cardiol. 2011; 148:331–36. 10.1016/j.ijcard.2009.11.025 20053466 PMC3108570

[34] Baba A, Yoshikawa T, Fukuda Y, Sugiyama T, Shimada M, Akaishi M, Tsuchimoto K, Ogawa S, Fu M. Autoantibodies against M2-muscarinic acetylcholine receptors: new upstream targets in atrial fibrillation in patients with dilated cardiomyopathy. Eur Heart J. 2004; 25:1108–15. 10.1016/j.ehj.2004.05.012 15231368

[35] Matsui S, Fu ML, Hayase M, Katsuda S, Yamaguchi N, Teraoka K, Kurihara T, Takekoshi N. Beneficial effect of muscarinic-2 antagonist on dilated cardiomyopathy induced by autoimmune mechanism against muscarinic-2 receptor. J Cardiovasc Pharmacol. 2001 (Suppl 1); 38:S43–49. 10.1097/00005344-200110001-00010 11811358

[36] Liu HR, Zhao RR, Zhi JM, Wu BW, Fu ML. Screening of serum autoantibodies to cardiac beta1-adrenoceptors and M2-muscarinic acetylcholine receptors in 408 healthy subjects of varying ages. Autoimmunity. 1999; 29:43–51. 10.3109/08916939908995971 10052684

[37] Fu ML. anti-M2 muscarinic receptor autoantibodies and idiopathic dilated cardiomyopathy. Int J Cardiol. 1996; 54:127–35. 10.1016/0167-5273(96)02589-2 8803676

[38] Fu LX, Magnusson Y, Bergh CH, Liljeqvist JA, Waagstein F, Hjalmarson A, Hoebeke J. Localization of a functional autoimmune epitope on the muscarinic acetylcholine receptor-2 in patients with idiopathic dilated cardiomyopathy. J Clin Invest. 1993; 91:1964–68. 10.1172/JCI116416 7683693 PMC288192

[39] Li H, Murphy T, Zhang L, Huang B, Veitla V, Scherlag BJ, Kem DC, Yu X. Β1-adrenergic and M2 muscarinic autoantibodies and thyroid hormone facilitate induction of atrial fibrillation in male rabbits. Endocrinology. 2016; 157:16–22. 10.1210/en.2015-1655 26517045

[40] Yu X, Riemekasten G, Petersen F. Autoantibodies against muscarinic acetylcholine receptor M_3_ in sjogren’s syndrome and corresponding mouse models. Front Biosci (Landmark Ed). 2018; 23:2053–64. 29772545 10.2741/4689

[41] Loebel M, Grabowski P, Heidecke H, Bauer S, Hanitsch LG, Wittke K, Meisel C, Reinke P, Volk HD, Fluge Ø, Mella O, Scheibenbogen C. Antibodies to β adrenergic and muscarinic cholinergic receptors in patients with chronic fatigue syndrome. Brain Behav Immun. 2016; 52:32–39. 10.1016/j.bbi.2015.09.013 26399744

[42] Scheibenbogen C, Loebel M, Freitag H, Krueger A, Bauer S, Antelmann M, Doehner W, Scherbakov N, Heidecke H, Reinke P, Volk HD, Grabowski P. Immunoadsorption to remove ß2 adrenergic receptor antibodies in chronic fatigue syndrome CFS/ME. PLoS One. 2018; 13:e0193672. 10.1371/journal.pone.0193672 29543914 PMC5854315

[43] Gunning WT 3rd, Kvale H, Kramer PM, Karabin BL, Grubb BP. Postural orthostatic tachycardia syndrome is associated with elevated g-protein coupled receptor autoantibodies. J Am Heart Assoc. 2019; 8:e013602. 10.1161/JAHA.119.013602 31495251 PMC6818019

[44] Elhusseiny A, Cohen Z, Olivier A, Stanimirović DB, Hamel E. Functional acetylcholine muscarinic receptor subtypes in human brain microcirculation: identification and cellular localization. J Cereb Blood Flow Metab. 1999; 19:794–802. 10.1097/00004647-199907000-00010 10413035

[45] Yamada M, Lamping KG, Duttaroy A, Zhang W, Cui Y, Bymaster FP, McKinzie DL, Felder CC, Deng CX, Faraci FM, Wess J. Cholinergic dilation of cerebral blood vessels is abolished in M_5_ muscarinic acetylcholine receptor knockout mice. Proc Natl Acad Sci USA. 2001; 98:14096–101. 10.1073/pnas.251542998 11707605 PMC61174

[46] Araya R, Noguchi T, Yuhki M, Kitamura N, Higuchi M, Saido TC, Seki K, Itohara S, Kawano M, Tanemura K, Takashima A, Yamada K, Kondoh Y, et al. Loss of M5 muscarinic acetylcholine receptors leads to cerebrovascular and neuronal abnormalities and cognitive deficits in mice. Neurobiol Dis. 2006; 24:334–44. 10.1016/j.nbd.2006.07.010 16956767

[47] Karczewski P, Hempel P, Kunze R, Bimmler M. Agonistic autoantibodies to the α_1_-adrenergic receptor and the β_2_-adrenergic receptor in Alzheimer’s and vascular dementia. Scand J Immunol. 2012; 75:524–30. 10.1111/j.1365-3083.2012.02684.x 22260197

[48] Lee YT, Lee HC, Hu CJ, Huang LK, Chao SP, Lin CP, Su EC, Lee YC, Chen CC. Periodontitis as a modifiable risk factor for dementia: a nationwide population-based cohort study. J Am Geriatr Soc. 2017; 65:301–05. 10.1111/jgs.14449 27685603

[49] Boivin-Jahns V, Jahns R, Boege F. Relevant effects of beta_1_-adrenoceptor autoantibodies in chronic heart failure. Front Biosci (Landmark Ed). 2018; 23:2146–56. 29772551 10.2741/4695

[50] Bornholz B, Hanzen B, Reinke Y, Felix SB, Boege F. Impact of common β1-adrenergic receptor polymorphisms on the interaction with agonistic autoantibodies in dilated cardiomyopathy. Int J Cardiol. 2016; 214:83–85. 10.1016/j.ijcard.2016.03.032 27057984

[51] Dorn JM, Genco RJ, Grossi SG, Falkner KL, Hovey KM, Iacoviello L, Trevisan M. Periodontal disease and recurrent cardiovascular events in survivors of myocardial infarction (MI): the western new york acute MI study. J Periodontol. 2010; 81:502–11. 10.1902/jop.2009.090499 20367093

[52] Eke PI, Page RC, Wei L, Thornton-Evans G, Genco RJ. Update of the case definitions for population-based surveillance of periodontitis. J Periodontol. 2012; 83:1449–54. 10.1902/jop.2012.110664 22420873 PMC6005373

[53] Armitage GC. Development of a classification system for periodontal diseases and conditions. Ann Periodontol. 1999; 4:1–6. 10.1902/annals.1999.4.1.1 10863370

