# Supplementary material for: Autoantibodies against M5-muscarinic and beta1-adrenergic receptors in periodontitis patients
Source: Aging (Albany NY). 2020 Aug 28;12(16):16609–20. doi: 10.18632/aging.103864 (PMC7485715; doi:10.18632/aging.103864)
Supplement: Supplementary Figures [file aging-12-103864-s001..pdf]

SUPPLEMENTARY FIGURES

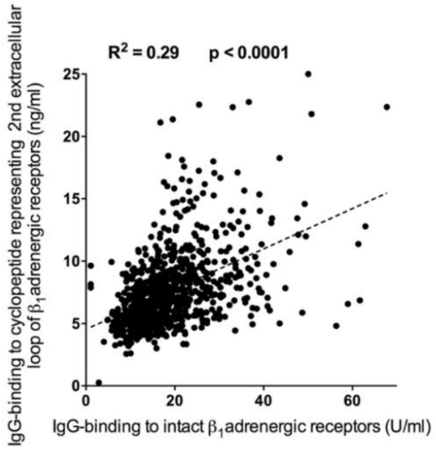

Supplementary Figure 1. Comparison of β1AR-Aabs measured by IgG-binding to the native intact receptor (CellTrend GmbH) or to a cyclic peptide providing a valid representation of the presumed pathogenic conformational auto-epitope within the second extracellular loop of the receptor associated with the active receptor conformation [23].

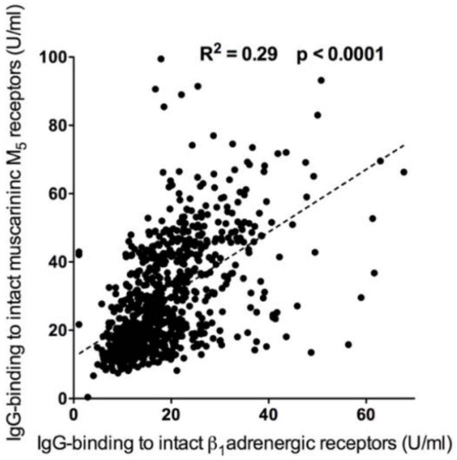

Supplementary Figure 2. Comparison of serum levels of β1AR-Aabs and M5R-Aabs in periodontitis patients. Both autoantibodies were measured by IgG-binding to the native intact receptor (CellTrend GmbH).
